# Body dissatisfaction and smartphone addiction: the mediation role of intrusive imagery and fear of negative evaluation

**DOI:** 10.3389/fpsyg.2023.1217220

**Published:** 2023-11-22

**Authors:** Shiyu Liu, Wen Hu, Yingkai Yang, Fahui Yang

**Affiliations:** ^1^Faculty of Psychology, Southwest University, Chongqing, China; ^2^Mindrun Educational Technology Co., Ltd., Shenzhen, China

**Keywords:** smartphone addiction, body dissatisfaction, intrusive imagery, fear of negative evaluation, serial mediation

## Abstract

This research aimed to examine the mediating effect of intrusive imagery and fear of negative evaluation on the connection between body dissatisfaction and smartphone addiction. 5,909 participants were included in the research, with 53.8% of the sample being female. The average age of the participants was 19.87 years, and their ages ranged from 18 to 32 years. All individuals who were recruited for the study successfully finished the Satisfaction and Dissatisfaction with Body Parts Scale, the Smartphone Addiction Scale, the Intrusive Imagery Scale, and the Fear of Negative Evaluation Scale. Mediational analysis indicated that, with age, gender and body mass index under control, body dissatisfaction was linked to smartphone addiction via (1) the mediating role of intrusive imagery, (2) the mediating role of fear of negative evaluation, and (3) the serial mediating role of intrusive imagery and fear of negative evaluation. Our study for the first time advanced our understanding that intrusive imagery and fear of negative evaluation could be serial mediators of the relationship between body dissatisfaction and smartphone addiction. However, the cross-sectional design prevented us from establishing causality between these variables, which could be better examined in future longitudinal studies.

## 1 Introduction

Smartphones have become increasingly popular worldwide due to their convenience and efficiency in performing various functions such as connecting with others or managing daily schedule via apps (Panova and Carbonell, 2018; Li et al., 2020). By 2020, the number of smartphone users had grown to approximately 4.78 billion, accounting for around 61.62% of the global population (Turner, 2020). However, along with their benefits, smartphones also bring about numerous issues including its overuse or even addiction (Çağan et al., 2014; Li et al., 2020), which have increasingly motivated relevant discussions and studies in recent years (Panova and Carbonell, 2018). Derived from the Fifth Edition of the Diagnostic and Statistical Manual of Mental Disorders (DSM-5), two categories of addiction emerged: substance addiction (such as alcohol and caffeine) and behavioral addiction (e.g., internet gaming) (Moattari et al., 2017). Building on this foundation, Kardefelt-Winther and his colleges outlined behavioral addiction through a dual framework: (1) the presence of noticeable impairment or distress directly stemming from the behavior and (2) its endurance over time (Li et al., 2023). While not included within DSM-5, behavioral patterns resembling behavioral addiction mentioned above have been verified in smartphone usage (Li et al., 2023). Broadly, smartphone addiction encompasses four primary elements: compulsive actions, tolerance, withdrawal, and impaired functionality (Lin et al., 2016). With the aim of assessing the severity of smartphone addiction within a relatively large population, certain tools have been crafted, including specialized mobile apps designated for identifying smartphone addiction (Lin et al., 2016). A few questionnaires, notably the Smartphone Addiction Scale, has shown commendable reliability and validity (Moattari et al., 2017). Comprising six primary dimensions (daily-life disturbance, positive anticipation, withdrawal, cyberspace-oriented relationship, overuse, and tolerance) (Kwon et al., 2013), SAS has been extensively employed in various adapted versions (Moattari et al., 2017). Nonetheless, delving into more objective assessments like the investigation of key brain regions linked to smartphone addiction remains a pertinent avenue for exploration (Lin et al., 2016). Given the planned large sample in our research, we elected to employ questionnaires as our preferred measurement tool. College students are especially vulnerable to smartphone addiction due to their immature self-control abilities, which means it is a challenge for college students to intentionally modify their behavior (Akın et al., 2015; Li et al., 2018, 2020), withstand the allure of smartphone addiction, manage their emotions, and engage in actions that align with their individual objectives. At the same time, they are faced high demand of smartphone use (e.g., to stay connected with families and friends) (Çağan et al., 2014; Gao et al., 2018; Li et al., 2020). Previous evidence shows smartphone addiction is associated with detrimental impact on mental and behavior problems, such as anxiety, depression, sleep disorders as well as hand dysfunction in the college students population (Çağan et al., 2014; Liu et al., 2020b). Therefore, it is essential to explore potential factors that have connection with smartphone addiction among college pupils so that prevention and intervention strategies could be better implemented.

Prior research has documented the consistent correlation between smartphone addiction and body dissatisfaction (Kang and Chee, 2019; Li et al., 2020; Mac Intyre et al., 2020; Yang et al., 2022). Body dissatisfaction refers to negative thoughts and feelings about one’s body due to the perceived discrepancy between one’s actual body image and ideal body image (Grogan, 2021; Yang et al., 2022). Body dissatisfaction has been identified as the proximal contributor to smartphone addiction. For example, based on a survey of 443 adolescents in Turkey, the extent of body dissatisfaction was positively associated with problematic smartphone use (Emirtekin et al., 2019). And based on another research of 1,036 teenagers conducted in China, body dissatisfaction scores were found positively associated with the risk of smartphone addiction (Liu et al., 2020b). As proposed by the cognitive-behavior model of internet addiction (Davis, 2001), adolescents who evaluate their body shape negatively are more susceptible to addiction due to their tendency to strategically highlight and only convey their most appealing qualities or seek validation from others while engaging in social networking site (Liu et al., 2020b; Yang et al., 2022). Positive responses, such as praise, received during this process are identified as a significant factor associated with ongoing smartphone use and an increased likelihood of smartphone addiction (Davis, 2001; Liu et al., 2020b). Similarly, in accordance with the compensatory satisfaction theory (Liu et al., 2016), adolescents dissatisfied with their real-world appearance might seek satisfaction in the virtual realm, selectively presenting positive aspects to create a desired image on social networking sites and garner approval (Kim and Lee, 2011; Abbasi et al., 2021). These positive feedback and fulfilled satisfaction are likely linked to reinforcing smartphone use, especially the use about social networking site, and elevating the risk of addiction (Xin et al., 2020; Liu et al., 2020a,b). Alternatively, based on compensatory smartphone use theory, individuals with averse personal experience due to body dissatisfaction may be driven to excessively and aimlessly smartphone use and it may be identified as a approach to alleviate their adverse affection (Wolniewicz et al., 2018; Zhang and Zhang, 2023). While smartphone use may temporarily alleviate body dissatisfaction, it could potentially reinforce such dissatisfaction through exposure to ideal body images and upward social comparisons (Liu et al., 2020b). However, to the best of our knowledge, reported longitudinal results and empirical evidence supporting this connection is still lacking so far. Based on existing theory and correlation results from previous research, we propose Hypothesis 1 (H1): Body dissatisfaction is positively correlated with smartphone addiction.

Furthermore, previous documents have suggested body dissatisfaction is potentially correlated with smartphone addiction through various mediating factors. Negative affectivity, like depression and anxiety, has been consistently identified as a proximal factor associated with smartphone addiction (Sohn et al., 2019; Yue et al., 2021; Zsido et al., 2021). Some prior documents have indicated a potential association between the fear of negative evaluation, which is a central component of social anxiety (Watson and Friend, 1969), and the development of smartphone addiction in college students (Wolniewicz et al., 2018; Wu, 2018; Li et al., 2020; Ali et al., 2021). Fear of negative evaluation was defined as apprehension about negative evaluations or judgment from others (Leary, 1983), eagerness to obtain positive evaluations, as well as avoidance of social evaluation situations (Watson and Friend, 1969; Utschig et al., 2010). Individuals who experience apprehension about receiving negative judgments from others may find social gatherings, especially in physical settings, to be more anxiety-inducing. Consequently, they might seek to fulfill their social demand via online platforms like chat room, which afford them greater control and flexibility (Ali et al., 2021). For instance, research suggests that email communication can alleviate anxiety and inhibition, offering enhanced preparation and control for individuals who fear negative evaluations (Keaten and Kelly, 2008). This motivation to fulfill social needs may be intricately linked with the development of smartphone addiction, as suggested by Uses and Gratifications Theory (Wolniewicz et al., 2018). Alternatively, it has been suggested that smartphone use serves as a maladaptive regulation strategy to alleviate negative feelings from real or anticipated negative evaluation (Kardefelt-Winther, 2014).

In addition, evidence have suggested a positive correlation between body dissatisfaction and fear of negative evaluation (Levinson and Rodebaugh, 2015; Pawijit et al., 2017). For example, in the research with a sample of 160 women, the score of fear of negative evaluation from self-report questionnaire is positively associated with level of body dissatisfaction (Levinson and Rodebaugh, 2015). Moreover, based on a follow-up longitudinal research that concentrated on undergraduate women, their degrees of body dissatisfaction could positively predict levels of fear of negative evaluation which were measured 6 and 12 months later (DeBoer et al., 2013). For college students, body shape is central to self-assessment (Maxwell and Cole, 2012), and those who have higher degrees of body dissatisfaction are more prone to harbor negative perceptions regarding their physical appearance and may consequently anticipate unfavorable evaluations from others in social contexts (Ahadzadeh et al., 2018). According to the evidence mentioned above, it is rational to propose Hypothesize 2a (H2a): fear of negative evaluation could play a mediating role between the link of body dissatisfaction on smartphone addiction.

Intrusive imagery refers to experience of perception accessing from memory rather than external sensory input, giving rise to the experience of “seeing with the mind’s eye” or “hearing with the mind’s ear” (Kosslyn et al., 2001). Intrusive imagery has a significant impact on individuals’ emotion and behavior (Holmes et al., 2008). It is characterized by recurrent and vivid images with multiple sensory modalities including visual, acoustic, and tactile elements, and often activated by situational or internal stimuli (Cili and Stopa, 2015). Although intrusive imagery has been recognized as a important transdiagnosis variable in the pathology of various mental disorders as well as problematic behaviors in recent times, such as depression, anxiety disorder, self-harm, suicidality, and addictive behaviors (Ji et al., 2019), previous research on intrusive psychological phenomena and behavioral addiction often studied intrusive thoughts as a whole, for example, Burnay and his colleagues indicated that a susceptibility to experiencing intrusive thoughts was correlated with increased engagement in Internet-related activities, such as someone overwhelmed with thoughts about their next online session may more possibly participate in online related activities (Burnay et al., 2015). Previous researchers, when studying intrusive thoughts, which were characterized by repetitive thoughts, images, or impulses that are unacceptable or unwanted (Clark, 2005; Thaiposri and Reece, 2022), overlooked the differences between verbal thoughts and mental imagery, even though both are included in intrusive thoughts. Among intrusive thoughts, mental imagery is distinctive from verbal thoughts through possessing various sensory modalities (Holmes et al., 2008; Holmes and Mathews, 2010). As previously noted, intrusive mental imagery and intrusive verbal thought were supported by two separate and distinct memory systems (McCarthy-Jones et al., 2012), namely the verbally accessible and situationally accessible memory systems, respectively (Hagenaars et al., 2010). The former is responsible for storing information pertaining to intrusive verbal thoughts with adequate conscious processing prior to encoding, whereas the latter charges information linked to intrusive imagery resulting from lower-level perceptual processing and the information contained therein is replete with a diverse range of sensory impressions (Hagenaars et al., 2010). Compared with intrusive verbal thoughts, imagery owns greater potential to amplify negative affection, as brain emotion processing is more responsive to mental imagery (McCarthy-Jones et al., 2012). Therefore, in our research, we tend to distinguish intrusive as a single critical factor from intrusive thought to explore its relationship with behavior addiction. Due to past research indicating similarities in characteristics and development processes between internet addiction and mobile phone addiction (Lin et al., 2016; Elhai et al., 2017), we speculate on the potential associations between mobile phone addiction and intrusive imagery by referencing the correlational evidence between internet addiction and intrusive thoughts mentioned above (Burnay et al., 2015), suggesting a possible association between the two. In line with the speculation of Burnay and his colleagues, intrusive mental images regarding an individual’s upcoming smartphone use session may potentially incite desires, leading to episodes of longing and manifesting as compulsive actions of smartphone use. Another conjectural elucidation is that the utilization of smartphones might serve as a method for diverting attention from intrusive imagery so that it could be under control and reduced. And in this case, the content of intrusive imagery may be unrelated with smartphone use (Burnay et al., 2015). Finally, the risk of smartphone addiction increases for the function for a long run.

While mature theoretical support is still lacking at present, some previous investigations have indirectly offered proof for the connection between body dissatisfaction and intrusive imagery. It is suggested that individuals with high level of body dissatisfaction have attention bias on body related information and feel more worried about their body shape (Tobin et al., 2018; Withnell et al., 2019; Talbot and Saleme, 2022). Some empirical evidence supported the relationship, for example, a research demonstrated attentional bias on fat and thin model images in a sample of 65 women with elevated degrees of body dissatisfaction through priming tasks (Withnell et al., 2019). Likewise, Talbot et al. have found men who was more dissatisfied with their body drawn more attention on body related information like ideal body shape or negative feature of their bodies through gaze tracking, target-dot and visual search task (Talbot and Saleme, 2022). Furthermore, when one’s attention is concentrated on body and appearance and feel more worried about their appearance, intrusive imagery would appear more frequently (Osman et al., 2004), and these intrusive imagery about participants’ appearance and body shape are often expected to be prompted by body related cues such as exposure to mirrors and appearance-related thoughts (Onden-Lim and Grisham, 2013). Despite these evidence aforementioned, there is a lack of direct evidence to support their correlation relationship. In our study, we propose Hypothesis 2b (H2b) to explore the possibility: intrusive imagery could play a mediating role between the link of body dissatisfaction on smartphone addiction.

In addition, considering the close association between intrusive imagery and negative affections (Cili and Stopa, 2015), as well as between negative affections and smartphone addiction mentioned above, it is plausible to speculate that intrusive imagery may incorporate another variable closely associated with adverse emotion to play a serial mediating effect in the connection between body dissatisfaction and smartphone addiction. Specially, individuals who reported they experienced more frequent intrusive imagery felt higher levels of social anxiety (Ashbaugh et al., 2019) and depression (Patel et al., 2007), which have been suggested to play critical roles in smartphone addiction, for smartphone use could help divert negative emotional content or substitute face-to-face interaction (Yue et al., 2021). Among these negative moods, some clinical intervention findings have lent support to the connection between intrusive imagery and fear of negative evaluation (Wild et al., 2008; Frets et al., 2014). For example, rescripting early memories linked to intrusive imagery significantly reduced scores of fear of negative evaluation reported by patients with social phobia (Wild et al., 2008). Similarly, based on a content analysis study, individuals with high-level social anxiety experienced intrusive images that manifest their fear of how they might appear to others and being criticized by others (Ashbaugh et al., 2019). Furthermore, as suggested by the research conducted by Osman and his colleagues, as people felt more worried and anxious about their body and appearance, they often reported more frequent intrusive imagery experience and higher level of fear of negative evaluation (Osman et al., 2004). Therefore, according to theories and evidence in introduction part, we speculate that people who feel dissatisfactory with their body may experience more frequent intrusive imagery and then feel higher degree of negative evaluation, smartphone addiction could probably be the reinforced result as the aforementioned various possible function of smartphone use. We proposed Hypothesis 2c (H2c): intrusive imagery and fear of negative evaluation might play a serial mediating role between the link of body dissatisfaction and smartphone addiction.

To summarize, the primary point of this research is to prove proposed hypothesizes in Chinese young adults as mentioned below:

*H1*: Body dissatisfaction is positively correlated with smartphone addiction.

*H2a*: Fear of negative evaluation could play a mediating role between the link of body dissatisfaction on smartphone addiction.

*H2b*: Intrusive imagery could play a mediating role between the link of body dissatisfaction on smartphone addiction.

*H2c*: Intrusive imagery and fear of negative evaluation might play a serial mediating role between the link of body dissatisfaction and smartphone addiction.

## 2 Method

### 2.1 Participants

Based on stratified random sampling, we selected four different universities located in relatively distant four provinces which is in distinct directions in China, including Chongqing, Guangdong, Shandong and Zhejiang. we contacted the counselors who was in charge with the monthly mental health condition survey in the mental healthcare center of each university. With their assistance of sending the link of questionnaires to WeChat groups, students receive the access to questionnaires and voluntarily filled out the online survey. Before completing the survey, participants had to read the brief explanation of our study purpose and the assurance for participants that data collection, storage, analyzing and reporting process would safeguard confidentiality and anonymity. If they did not agree to the terms, the questionnaire was automatically terminated. The Ethical Committee for Scientific Research of authors’ affiliated university approved research methodology and data management processes in keeping with ethical principles.

Finally, participants were 5,909 college students (53.8% females based on gender dichotomy). Participants in our study had a mean age of 19.87 (*SD* = 1.73, range = 18–32). Furthermore, their mean BMI (body mass index) was 20.39 (*SD* = 2.98, range = 14.42–37.34). Among the participants, 76.7% (*n* = 4,529) were classified as either underweight or of normal weight, with a BMI < 24 kg/m^2^. By contrast, 6.9% (*n* = 408) were overweight, with a BMI ranging from 24 to 27.99 kg/m^2^, while 2.3% (*n* = 137) were obese, with a BMI ≥ 28 kg/m^2^. Nevertheless, it should be noted that the BMI data for 835 participants were missing due to their failure to indicate their height or weight.

### 2.2 Measures

#### 2.2.1 Body dissatisfaction

In order to assess the degree of body dissatisfaction among participants, the Satisfaction and Dissatisfaction with Body Parts Scale (Berscheid et al., 1973) was employed. This scale comprises 9 items that prompt respondents to rate their level of satisfaction with nine different body parts (including waist, thighs, hips, crotch, legs, height, figure, full body shape, abdomen) with a 5-point Likert-type scale, in which 1 suggests extreme satisfaction and 5 suggests extreme dissatisfaction. A composite mark was calculated by summing the scores of all items, wherein greater marks were suggestive of increased levels of body dissatisfaction among individuals. This measurement scale has been applied to Chinese participants, demonstrating robust construct validity and favorable internal consistency (Jackson and Chen, 2011; Sukamto et al., 2013). Among college students, its Cronbach’s alpha coefficient reached 0.915, indicating satisfactory reliability (Sukamto et al., 2013). The internal consistency of the scale was assessed using the Cronbach coefficient and yielded a value of 0.95 in the current study.

#### 2.2.2 Intrusive imagery

Intrusive imagery was measured with the Intrusive Visual Imagery Scale (McCarthy-Jones et al., 2012), which was developed to measure the tendency to experience intrusive imagery (e.g.: “there are images that come to mind that I cannot erase.”; “I find it hard to sleep as images keep coming into my head.”) This 10-item used a 5-point Likert-type scale with a range from “strongly disagree” (1) to “strongly agree” (5). These items, when combined, produced an index that reflected the participants’ propensity to experience intrusive imagery, with higher composite scores indicating more experience of intrusive imagery. The Intrusive Visual Imagery Scale draws inspiration from the White Bear Suppression Inventory (Wegner and Zanakos, 1994) and the Thought Control Ability Questionnaire (Luciano et al., 2005). Unlike the previous focus on prospective imagery alone, as evaluated by the Impact of Future Events Scale in prior studies (Deeprose and Holmes, 2010), this adapted scale aims to comprehensively capture the broader spectrum of intrusive imagery experiences. The Intrusive Imagery Scale had been used with British young adults’ participants and the Cronbach’s alpha coefficient was 0.89 (McCarthy-Jones et al., 2012). The reliability analysis conducted in the current investigation yielded a Cronbach’s alpha coefficient of 0.97.

#### 2.2.3 Fear of negative evaluation

Fear of negative evaluation was measured using the Fear of Negative Evaluation Scale (FNE) (Watson and Friend, 1969). This 30-item assesses the fear of receiving negative evaluations from others (Wang et al., 2015) (e.g.: “I worry about what other people will think of me even when I know it does not make any difference.”; “I am frequently afraid of other people noticing my shortcomings.”). Each question is scored 1 (not at all characteristic or true of me) to 5 (extremely characteristic or true of me) with lower scores indicating the peace of mind about others’ evaluations while higher scores indicating the tendency to avoid potentially threatening social comparisons, feel more nervous in situations with social appraisals and make more effort to increase approval or avoid disapproval for which they will felt worse when receiving it (Heimberg et al., 1988). This questionnaire has been applied to Chinese college students, demonstrating psychometric properties (Ali et al., 2021). The reliability analysis conducted in the current investigation produced a Cronbach’s alpha coefficient of 0.87.

#### 2.2.4 Smartphone addiction

The present study sought to assess smartphone addiction using the Smartphone Addiction Scale-Short Version (Kwon et al., 2013). This instrument comprises of a 10-item scale, each scored on a 6-point Likert-type response format anchored at 1 (strongly disagree) to 6 (Strongly Agree). The summation of scores across all items was used to generate an index indicating the degree of susceptibility to smartphone addiction, where higher scores indicate greater risk for this phenomenon (e.g.: “Missing planned work due to smartphone use”; “I will never give up using my smartphone even when my daily life is already greatly affected by it.”). This measurement has been widely used with Chinese university students’ sample (Zhang et al., 2022; Yue et al., 2023), demonstrating its suitability as a reliable tool for assessing smartphone addiction among Chinese university students (Zhao et al., 2022). The reliability analysis conducted in the current investigation yielded a Cronbach’s alpha coefficient of 0.95.

#### 2.2.5 Covariates

We identify BMI, age, gender as covariates considering they are important factors related with body dissatisfaction and smartphone addiction based on previous research (Yang et al., 2019; Chen et al., 2020). Participants provided self-reported data regarding weight, height, age and gender. BMI was then calculated using the conventional formula of weight in kilograms divided by height in meters squared, yielding a score expressed in units of kg/m^2^.

### 2.3 Data analysis

In the current study, we employed SPSS 24.0 to conduct analysis for common method bias, descriptive statistics, correlation analyses and independent samples *t*-test. The potential mediating roles of intrusive imagery and fear of negative evaluation in the connection between body dissatisfaction and smartphone addiction were examined through mediating effect analyses performed via R version 3.6.2 and the lavaan R package edition 0.6–9. While formulating the model code, we designated body dissatisfaction as the independent variable, smartphone addiction as the dependent variable, and identified intrusive imagery and fear of negative evaluation as mediators. Ultimately, we constructed a comprehensive multiple mediation model comprising both parallel mediators and serial mediation. Moreover, covariates were controlled for in all mediation analyses, and missing values of age and BMI were handled through full information maximum likelihood. Following that, in order to assess the significance of the multiple mediation model, we applied the bootstrap resampling technique in our code with 5,000 bootstrap samples to further evaluate the mediating effects and a mediation was determined present when the 95% confidence interval for the mediation index did not encompass 0.

## 3 Results

### 3.1 Common method bias analysis

To assess the potential for common method bias in this study, all data were gathered through self-report questionnaires. Common method bias test was conducted and revealed that the first factor accounted for 23.84% of the total variance, falling below the recommended threshold of 50% (Podsakoff et al., 2003). Based on this finding, it can be concluded that common method bias is not a significant concern in this study.

### 3.2 Preliminary analyses

Table 1 displays the descriptive statistics and correlation matrix of the variables examined in the present study. Results showed age was significantly correlated with body dissatisfaction (*r* = −0.04, 95% CI = [−0.06,−0.01], *p* < 0.001), intrusive imagery (*r* = −0.06, 95% CI = [−0.08,−0.03], *p* < 0.001), fear of negative evaluation (*r* = −0.1, 95% CI = [−0.01,−0.31], *p* < 0.001) and smartphone addiction (*r* = −0.05, 95% CI = [−0.08,−0.02] *p* < 0.001); gender was significantly associated with body dissatisfaction (*r* = −0.20, 95% CI = [−0.23,−0.18], *p* < 0.001), intrusive imagery (*r* = −0.09, 95% CI = [−0.12,−0.06], *p* < 0.001), fear of negative evaluation (*r* = −0.16, 95% CI = [−0.19,−0.14], *p* < 0.001) and smartphone addiction (*r* = −0.18, 95% CI = [−0.2,−0.15], *p* < 0.001); BMI was significantly correlated with body dissatisfaction (*r* = 0.20, 95% CI = [0.18,0.23], *p* < 0.001), fear of negative evaluation (*r* = −0.04, 95% CI = [−0.07,−0.01], *p* < 0.001)and smartphone addiction (*r* = −0.03, 95% CI = [−0.06,−0.003], *p* < 0.001). Then we performed independent samples *t*-test to ensure whether the questionnaires differed depending on different genders. Results showed that scores of body dissatisfaction (*t* = 16.60, *p* < 0.01), fear of negative evaluation (*t* = 12.66, *p* < 0.01) and smartphone addiction (*t* = 13.66, *p* < 0.01) of the participants were statistically different between participants with different genders (male = 1, female = 0), while scores of intrusive imagery were no significant difference between them (*t* = 6.56, *p* = 0.34).

**Table 1:** Correlations, means, standard deviations and ranges of investigated variables.

Variables	*M* (*SD*)	Range	1	2	3	4	5	6	7
1 Age	19.87 (1.73)	[18, 32]	1						
2 Gender	——	——	——	1					
3 BMI	20.39 (2.98)	[14.42, 37.34]	0.005	0.33^**^	1				
4 Body dissatisfaction	16.66 (8.39)	[0, 36]	0.04^**^	−0.20^**^	0.20^**^	1			
5 Intrusive imagery	17.19 (8.77)	[10, 50]	−0.06^**^	−0.09^**^	−0.02	0.22^**^	1		
6 Fear of negative evaluation	78.77 (15.81)	[21, 137]	−0.10^*^	−0.16^**^	−0.04^**^	0.28^**^	0.41^**^	1	
7 Smartphone addiction	28.24 (11.65)	[10, 60]	−0.05^*^	−0.18^**^	- 0.03^*^	0.23^**^	0.44^**^	0.49^**^	1

*M*: mean; *SD*: standard deviation; BMI: body mass index. *p < 0.05, **p < 0.01.

The correlations between gender and the other variables were examined using point-biserial correlations.

Moreover, as expected, body dissatisfaction was positively correlated with intrusive imagery, *r* = 0.22, 95% CI = [0.19,0.25], *p* < 0.001, fear of negative evaluation, *r* = 0.28, 95% CI = [0.25,0.31], *p* < 0.001, and smartphone addiction, *r* = 0.23, 95% CI = [0.21,0.27], *p* < 0.001, which supports H1. Intrusive imagery was positively associated with fear of negative evaluation, *r* = 0.41, 95% CI = [0.39,0.43], *p* < 0.001 and smartphone addiction*, r* = 0.44, 95% CI = [0.42,0.46], *p* < 0.001. In addition, fear of negative evaluation was positively associated with smartphone addiction, *r* = 0.49, 95% CI = [0.46,0.51], *p* < 0.001.

Taking into account the relation proved by previous research and given that the present findings have demonstrated notable correlations between age, gender, BMI, and the variables within the planned serial mediating model, we introduced age, gender, and BMI as covariates. This step was taken to safeguard against any potential confounding effects these factors might have on our results.

### 3.3 Mediating model analyses

**Figure 1:**
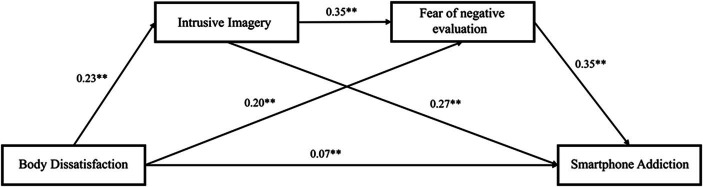
Multiple mediation models predicting smartphone addiction from body dissatisfaction, intrusive imagery and fear of negative evaluation. The standardized path coefficients have been utilized in the analysis. **p < 0.01.

Table 2 and Figure 1 present the findings of the multiple mediation analysis. Parallel mediating analysis indicated that intrusive imagery plays a significant mediating role in the relationship between body dissatisfaction and smartphone addiction (indirect effect =0.06, *p* < 0.001, 95% CI = [0.05–0.07]), which supports H2b. The indirect effects through intrusive imagery accounted for 27.6% (percentage mediated = 0.28, 95% CI = [0.23 0.32], *p* < 0.001) of the variances explained in smartphone addiction by body dissatisfaction.

**Table 2:** Summary of indirect effects from body dissatisfaction to smartphone addiction.

	Coefficient	SE	95% CI	*p*
Indirect effects (via mediators)				
BD → IM → SA	0.063	0.005	0.053, 0.072	<0.001
BD → FNE → SA	0.071	0.005	0.061, 0.080	<0.001
BD → IM → FNE → SA	0.028	0.002	0.024, 0.032	<0.001

SE = standard error; BD = body dissatisfaction; IM = intrusive imagery; SA = smartphone addiction; FE = fear of negative evaluation.

Similarly, fear of negative evaluation, as another parallel mediator, plays a significant mediating role in the relationship between body dissatisfaction and smartphone addiction (indirect effect = 0.07, *p* < 0.001, 95% CI = [0.06–0.08]), which supports H2a. The indirect effects through fear of negative evaluation accounted for 30.7% (percentage mediated = 0.31, *p* < 0.001, 95% CI = [0.26, 0.36]) of the variances explained in smartphone addiction by body dissatisfaction.

Besides, intrusive imagery and fear of negative evaluation play serial mediating roles in the relationship between body dissatisfaction and smartphone addiction (serial indirect effect = 0.03, *p* < 0.001,95% CI = [0.02–0.03]), which supports H2c. The serial indirect effects accounted for 12.4% (percentage mediated = 0.12, *p* < 0.001, 95% CI = [0.10, 0.14]) of the overall indirect effects.

Briefly, outcomes showed that intrusive imagery and fear of negative evaluation sequentially mediated the relationship between body dissatisfaction and smartphone addiction.

## 4 Discussion

The main objective of this research was to examine the potential association between body dissatisfaction and smartphone addiction among university students in China, while also investigating the mediating effects of intrusive imagery and fear of negative evaluation on the association. In line with previous studies (Emirtekin et al., 2019), our data demonstrated that body dissatisfaction was positively correlated with smartphone addiction and this relationship was mediated by fear of negative evaluation. Prio research has recognized the strong connection between sociocultural factors and body dissatisfaction, which serves as reflections of societal value judgments (Behravan and Steins, 2023). Body dissatisfaction is common not only in Western developed countries but also in contemporary China (Chen et al., 2007), where adolescent females report higher levels of body dissatisfaction compared to males (Xu et al., 2010), in line with the findings of this research. Similar with Western countries, young Chinese men prefer muscularity while thin body shape is regarded as the ideal among young females (Xu et al., 2010). As the internet and smartphones have become prevalent in China, individuals are highly susceptible to media influence. Studies reveal that young females often experience media-induced pressure to lose weight (Xu et al., 2010), with thin-ideal internalization identified as a mediation through which social culture impacts body image concerns (Chen et al., 2007). Additionally, males are also affected by social media and are more inclined to engage in muscle-building fitness activities (Xu et al., 2010). These sociocultural factors offer us possibility to explore the relationship between body dissatisfaction and smartphone addiction in this research. Simultaneously, for young individuals in China, peer and relative relationships play pivotal roles in shaping body dissatisfaction (Xu et al., 2010). This may be attributed to collectivist culture in China, where evaluations from others, especially close relationships, significantly impact individuals. This cultural aspect could also explain the study’s findings, indicating the connection between the fear of negative evaluations and body dissatisfaction, among other factors, highlighting a cultural specificity.

Additionally, our study has first demonstrated body dissatisfaction has the potential to impact smartphone addiction by means of the mediating effect of intrusive imagery and the serial mediating effect of intrusive imagery and fear of negative evaluation. To avoid misleading, we would like to emphasize two points first. Firstly, due to the measurement questionnaire for smartphone addiction in this study not including specific platform usage information, and the lack of consensus in the current concept of smartphone addiction regarding platform usage – for instance, Zhang and his colleagues suggested that mobile addiction often lacked a specific purpose and was characterized by the use of multiple platforms (Zhang and Zhang, 2023), while other research found that specific functions within mobile phones (such as photo-editing apps and social networking sites) were closely related factors in mobile phone addiction (Liu et al., 2020b). Therefore, in the discussion section, we will strive to include as many possibilities as possible regarding mobile addiction to provide more information for future research. Similarly, our measurement of intrusive imagery only involves the frequency of imagery occurrence and does not delve into the content of the imagery. So, if the discussion involves the content of imagery, we will also explore as many possibilities as possible.

As previously documented, fear of negative evaluation has emerged as a mediator in the relationship between body dissatisfaction and smartphone addiction. For example, Emirtekin et al. (2019) suggested that social anxiety, including fear of negative evaluation, mediated the influence of body dissatisfaction on smartphone addiction among Turkey adolescents. In line with these previous investigations, our study supported the pattern among Chinese university students. Individuals who exhibit elevated levels of body dissatisfaction are more susceptible to unfavorable assessments, ultimately leading to the development of apprehension toward criticism from others (Ahadzadeh et al., 2018). In addition, they always hold intense craving for receiving praise as evidence of external validation to confirm they are maintaining appearances on the same footing as social ideals (Pawijit et al., 2017). Therefore, according to theory of Uses and Gratifications (Wolniewicz et al., 2018), smartphone use probably serves as an adaptive way to compensate the craving and earn temporary satisfaction through selectively focusing or presenting their bright side of bodies on social networking site (Davis, 2001; Li et al., 2020; Liu et al., 2020b). Similarly from a specific purpose perspective, smartphone use might also compensate social interaction demand held back for the fear of receiving negative evaluations in real life (Bolle, 2014), as social interaction in smartphone like chatroom is more flexible and controllable (Ali et al., 2021). Meanwhile, based on Compensatory Internet Use Theory (Kardefelt-Winther, 2014), from a general smartphone use perspective, smartphone may also play its role in distracting oneself from distressing emotions due to fear of negative evaluation in social evaluation settings (Chen and Drummond, 2008), serving as a maladaptive affection regulation approach (Kardefelt-Winther, 2014). Hence, it is crucial to acknowledge that effective measures for preventing and managing smartphone addiction must encompass approaches that specifically address the regulation and motivation of behavioral patterns concerning negative affections.

Our research also paved the way to examine the probable mediating effect of intrusive imagery in the association between body dissatisfaction and smartphone addiction among Chinese university pupils. Previous documents have proved the impact of intrusive imagery among different mental disorders such as body dysmorphic disorder, posttraumatic stress disorder, social anxiety and depression (Kadriu et al., 2019). Based on the transdiagnostic common occurrences of intrusive images, our research suggested intrusive imagery might also play its role in smartphone addiction, which has not yet been explored by others. The incorporation of intrusive imagery within the context of smartphone addiction may potentially serve as a novel point in the cognitive conceptualization of its onset and maintenance, thus presenting a new avenue for alternative intervention strategies. Based on our results, we speculate that individuals with greater levels of body dissatisfaction might focus more attention on body or appearance related information (Tobin et al., 2019), and in this context they are probably more vulnerable for cues to induce intrusive imagery (Onden-Lim and Grisham, 2013). Some previous evidence lent support to this inference, for instance, based on a systematic review, results from eleven studies with eye-tracking method indicate that individuals exhibiting elevated levels of body dissatisfaction tend to allocate increased attention to stimuli associated with appearance when compared to control groups (Rodgers and DuBois, 2016); likewise, other research with different paradigm indicates that people experiencing higher levels of body dissatisfaction exhibit quicker responses to probes that replaced appearance related stimuli compared to other stimuli. This suggests a heightened attentional bias toward appearance-related stimuli in contrast to individuals with lower levels of body dissatisfaction (Rodgers and DuBois, 2016). Moreover, when individuals focus their attention on their bodies and appearance and are more preoccupied with their looks, it is anticipated that intrusive imagery will be triggered more frequently by cues related to their bodies, such as exposure to mirrors and thoughts related to appearance (Osman et al., 2004; Onden-Lim and Grisham, 2013). Additionally, they may become more sensitive to priming cues as they tend to interpret ambiguous stimuli as related to appearance or the body, as indicated by visual dot-probe evidence (Rosser et al., 2010).

Moreover, smartphone use might be reinforced as its function to cope with negative experience derived from intrusive imagery through general and purposeless smartphone use with multiple apps (Kardefelt-Winther, 2014; Zhang and Zhang, 2023), as the promotion of intrusive imagery may lead to distressing emotion (Brewin et al., 2010; Cili and Stopa, 2015). Specially, the research conducted by Kadriu et al. (2019) indicated that contents of intrusive imagery reported by individuals with high body dissatisfaction scores focused on body checking and negative self. Similarly, individuals diagnosed with bulimia nervosa (BN) demonstrated an association between intrusive mental imagery and recollections pertaining to unfavorable remarks concerning their weight or physical appearance (Kadriu et al., 2019). These negative and vivid contents would bring negative experience to individuals and smartphone use tend to be reinforced despite its negative addictive outcomes (Kardefelt-Winther, 2014; Cili and Stopa, 2015). Furthermore, from the perspective of more specific function of smartphone use, according to Self-Memory System model (Conway et al., 2004; Conway, 2005), these trauma experience in form of intrusive imagery might be identified as a threat to self-coherence (Cili and Stopa, 2015), while intrusive imagery also represents goals to avoid the perceived threat (Holmes and Mathews, 2010). To avoid the negative self-belief due to negative experiences in the intrusive imagery, individuals could take actions to extend the discrepancy between intrusive imagery and individuals’ actual state (Cili and Stopa, 2015), like posting the edited body photos on the website to receive approval. Thus, the reinforcement of habitual behaviors may ultimately result in an elevated propensity toward smartphone addiction over an extended period. Supporting the inference is the evidence that Arpaci (2021) had suggested approval seeking was a positive, and significant factor contributing to smartphone addiction among 660 mobile users in Turkey.

Our data suggest that intrusive imagery could also indirectly influence smartphone addiction through fear of negative evaluation. Consistent with previous literature, our outcome demonstrates the critical impact of intrusive imagery on affection (Cili and Stopa, 2015). For example, Cili and Stopa had reported that intrusive imagery could evoke negative emotions such as anxiety, fear, guilt and shame in social situations (Spurr and Stopa, 2003; Holmes and Mathews, 2010; Cili and Stopa, 2015). Similarly, as proposed by Hirsch et al. (2006), individuals may heightened anxiety and worry about bad performance during public speech if they have been required to rehearse negative images about themselves beforehand. Our study has broadened the range of emotions affected by intrusive imagery to include fear of negative evaluation, which is another crucial affective response associated with social circumstances. As we have noted above, intrusive imagery could evoke individuals’ negative self-beliefs about body, which probably increases their fear of receiving criticism from others (Ahadzadeh et al., 2018). Moreover, from the perspective of more specific smartphone use, to cope with the distressing psychological experience including threat to coherent self and apprehension, smartphone use may be reinforced since its role to expand discrepancy through seeking approval or reassurance (Cili and Stopa, 2015), as well as substitute the face-to-face communication avoided by individuals due to fear of receiving negative evaluations and compensate their social interaction demands (Bolle, 2014; Wolniewicz et al., 2018). In addition, from the perspective of more general smartphone use, smartphone use may be also reinforced for its functions to get temporal relief via distracting attention from distressing affection (Kardefelt-Winther, 2014).

By and large, our research expands upon prior studies by examining the unique mediating influence of intrusive imagery and the serial mediating effect of intrusive imagery as well as fear of negative evaluation in the connection between body dissatisfaction and smartphone addiction in the sample of Chinese university pupils. Furthermore, these discoveries hold significant clinical implications regarding the prevention and treatment of smartphone addiction. Given the impact of intrusive imagery on emotion and subsequent response behavior shown in our study, it could be proposed that gaining a more profound comprehension of mental imagery-related dysfunctions holds promise for advancing the conceptualization of smartphone addiction, thereby facilitating informed treatment decision-making and promoting the development of innovative imagery-focused treatments (e.g., positive image training focused on body shape).

## 5 Limitations and future research directions

It is essential to acknowledge that the research has certain limitations. First of all, our study, despite having a large sample size, was limited to college students, which may restrain the generalizability of the research findings to the wider population. In order to improve the generalizability of the findings, it is crucial that future study involve a more varied spectrum of people from different educational and cultural backgrounds. Additionally, sampling population of different ages, like teenagers, would probably yield useful information for future studies. Secondly, the cross-sectional design precludes the inference of causal relationships among the variables examined in our study (body dissatisfaction, intrusive imagery, fear of negative evaluation and smartphone addiction). While some of the research variables selected in this study, such as body dissatisfaction and smartphone addiction, have accumulated substantial supporting correlational evidence, two novel and significant factors, intrusive imagery and negative evaluation fear, have yet to demonstrate direct correlations with body dissatisfaction and smartphone addiction, despite indirect indications from certain research results or theories. In order to provide further evidence support for subsequent experimental research, we initially chose to conduct a multiple mediation analysis of these four variables in a large sample. Simultaneously, incorporating all four variables into one experimental design would complicate the manipulation of variables and the analysis of various effects in the results, making it challenging to present a clear and persuasive experimental design and outcomes. Therefore, in this study, we did not opt for an experimental research design. Henceforth, based on the finding in our research, it is advisable for forthcoming research to conduct experimental and longitudinal studies in order to examine the temporal and causal connections between aforementioned factors. Past research has established a series of precedents for using experimental methods to investigate the causal relationships among our research variables and related factors, such as the study conducted by Silva and Steins, they controlled the content of internet or smartphone use, whether the participants were exposed to diverse body types or a singular idealized body type, to investigate the impact of social media exposure on individuals’ body dissatisfaction (Castellanos Silva and Steins, 2023). Similarly, another study placed smartphone usage in different life-related contexts to explore potential factors influencing smartphone addiction (Ben-Yehuda et al., 2016). Subsequent experiments can reference these experimental designs to further explore the causal relationships among the investigated variables. Thirdly, we utilized self-report measures to collect all data, therefore, how subjectivity might affect the participants’ responses should be considered (e.g., memory recall, social desirability). Future studies could take advantage of incorporating self-reporting approaches with more objective means to quantify the use of smart phones (e.g., monitoring software, social desirability scales) to reduce the variation of common methods (Lin et al., 2016; Herrero et al., 2019). Finally, we collected limited information about our investigated variables. As for intrusive imagery, there are more valuable details to be explored, like the impact of intrusive imagery experience variance over time and the qualitative analysis based on reported content. Therefore, future investigations could improve the understanding of intrusive imagery to enhance the pattern in this study. As for smartphone addiction, the questionnaire we used did not inquire about specific purposes and platform information during smartphone usage (Kwon et al., 2013), yet these more detailed insights are crucial for exploring the pathways to smartphone addiction. Therefore, future research should consider including these details in measurements to obtain more accurate results in the investigation of the formation process of smartphone addiction. Moreover, our research holds potential for expansion by exploring other factors like significant demographic variables (e.g., socio-economic status, cultural background) and alternative mediators or moderators (e.g., sexual orientation, self-esteem) pertaining to the correlation between body dissatisfaction as well as smartphone addiction. It’s worth mentioning that body dissatisfaction is a variable closely connected with social and cultural factors, so it is essential to include demographic variables about the social and cultural background in future research.

## 6 Conclusion

In summary, our investigation highlights the essentiality of incorporating the mediating factors of intrusive imagery and fear of negative evaluation to comprehensively explicate the connection between body dissatisfaction and smartphone addiction in the Chinese university students’ population. The results further reveal that strategies aimed at addressing intrusive imagery and negative affect associated with the fear of negative evaluation may prove efficacious in ameliorating the harmful impact of body dissatisfaction on smartphone addiction.

## Data availability statement

The raw data supporting the conclusions of this article will be made available by the authors, without undue reservation.

## Author contributions

SL, WH, and FY contributed to development and design of methodology and completed data gathering. SL and YY conducted statistical analysis and results interpretation. SL composed the first draft of manuscript. WH and YY reviewed the manuscript. All authors have reviewed and approved the final version of the text.
